# Comparison of intestinal flora between patients with chronic and advanced *Schistosoma japonicum* infection

**DOI:** 10.1186/s13071-022-05539-6

**Published:** 2022-11-07

**Authors:** Chen Zhou, Junhui Li, Chen Guo, Zhaoqin Zhou, Zhen Yang, Yu Zhang, Jie Jiang, Yu Cai, Jie Zhou, Yingzi Ming

**Affiliations:** 1 Transplantation Center, Engineering and Technology Research Center for Transplantation Medicine of National Health Comission, The Third Xiangya Hospital, Central South University, Changsha, Hunan China; 2 Schistosomiasis Control Institute of Hunan Province, Yueyang, Hunan China

**Keywords:** *Schistosoma. japonicum*, Gut microbiota, Biomarker, Chronic *S. japonicum* infection, Advanced *S. japonicum* infection

## Abstract

**Background:**

*Schistosoma japonicum* infection is an important public health problem, imposing heavy social and economic burdens in 78 countries worldwide. However, the mechanism of transition from chronic to advanced *S. japonicum* infection remains largely unknown. Evidences suggested that gut microbiota plays a role in the pathogenesis of *S. japonicum* infection. However, the composition of the gut microbiota in patients with chronic and advanced *S. japonicum* infection is not well defined. In this study, we compared the composition of the intestinal flora in patients with chronic and advanced *S. japonicum* infection.

**Methods:**

The feces of 24 patients with chronic *S. japonicum* infection and five patients with advanced *S. japonicum* infection from the same area were collected according to standard procedures, and 16S rRNA sequencing technology was used to analyze the intestinal microbial composition of the two groups of patients.

**Results:**

We found that alteration occurs in the gut microbiota between the groups of patients with chronic and advanced *S. japonicum* infections. Analysis of alpha and beta diversity indicated that the diversity and abundance of intestinal flora in patients with advanced *S. japonicum* infection were lower than those in patients with chronic *S. japonicum* infection. Furthermore, *Prevotella* 9, *Subdoligranulum, Ruminococcus torques*, *Megamonas* and *Fusicatenibacter* seemed to have potential to discriminate different stages of *S. japonicum* infection and to act as biomarkers for diagnosis. Function prediction analysis revealed that microbiota function in the chronic group was focused on translation and cell growth and death, while that in the advanced group was concentrated on elevating metabolism-related functions.

**Conclusions:**

Our study demonstrated that alteration in gut microbiota in different stages of *S. japonicum* infection plays a potential role in the pathogenesis of transition from chronic to advanced *S. japonicum* infection. However, further validation in the clinic is needed, and the underlying mechanism requires further study.

**Supplementary Information:**

The online version contains supplementary material available at 10.1186/s13071-022-05539-6.

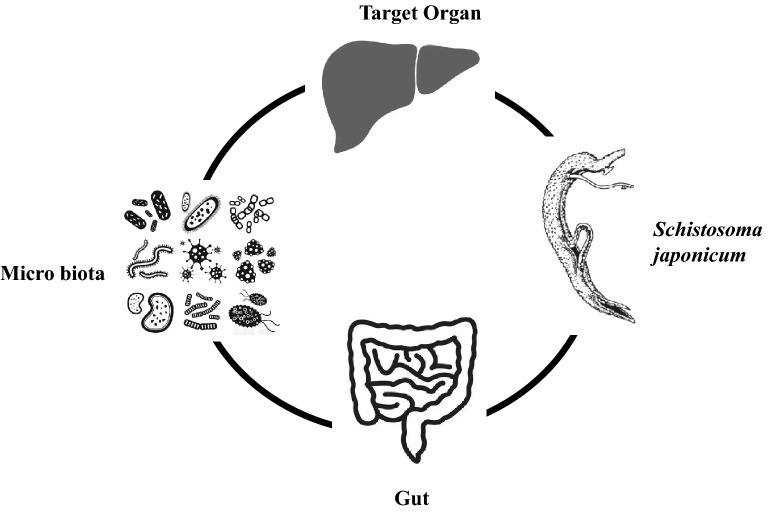

## Background

*Schistosoma japonicum* infection is one of the most prevalent neglected tropical diseases, affecting more than 240 million people in 78 countries worldwide and claiming 250,000 lives annually [1]. This progressive and debilitating parasitic disease imposes a heavy social and economic burden on people in pandemic areas. There are five main species of *Schistosoma* that can infect humans, including *S. japonicum, S. mansoni, S. haematobium, S. intercalatum* and *S. mekongi.* However, *S. japonicum* is the only *Schistosoma* species responsible for human infection in China, particularly in 12 provinces south of the Yangtze River [2]. Although China has achieved great progress in controlling *S. japonicum* infection, reducing infected cases by > 99.0%, it remains a public health problem in about 140 counties.

*Schistosoma japonicum* infection occurs when people come in contact with contaminated water. It is classified into three distinct stages according to the disease progression, including acute, chronic and advanced *S. japonicum* infection [3]. Patients with advanced infection often suffer from many serious complications such as portal hypertension [4], ascites [5], splenomegaly [6], etc., which lead to physical weakness, loss of labor and even death [7]. Although advances have been achieved on the road to eliminating *S. japonicum* infection, it has several big challenges in the last mile to conquer *S.japonicum* infection. There is no effective vaccine in the clinic, and development of new drugs is needed as only praziquantel is available [8]. In addition, praziquantel seems ineffective at preventing chronic *S. japonicum* infection from developing into the advanced stage [9]. Also, the mechanism of transition from chronic to advanced stages is not well defined. Naturally, it is hard to evaluate the risk and perform prognostic analysis on this progress. Thus, further study is needed in this field, particularly on the underlying mechanism in the transition from chronic to advanced *S. japonicum* infection.

Evidence has shown that the gut microbiota plays an important role in maintaining human health [10] and is also involved in various diseases [11]. High-quality data from the US Human Microbiome Project (HMP) [12], European Metagenomics of the Human Intestinal Tract (MetaHIT) [13] and other studies have demonstrated that normal gut microbiota can influence human health by affecting gene expression. Evidences also showed that gut microbiota has an impact on human metabolism and immunity [14–16]. Particularly *Bacteroides thetaiotaomicron* can promote the efficiency of lipid hydrolysis [15], while members of the genus *Bacteroides* can reduce blood lipids and modulate immunity by synthesizing conjugated linoleic acid (CLA) [16]. The occurrence and progression of various diseases often lead to changes in the composition of the gut microbiota, and alterations in gut microbiota also impact the pathogenesis of different diseases [11]. Imbalance in the gut microbiota is often related to an increased risk of various diseases; for example, gut microbiota disrupted by antibiotics seems to increase the risk of new-onset Crohn's disease [17]. Also, alterations in gut microbial composition and diversity have often been observed in patients with gastric cancer, liver cancer and many other tumors [18]. As gut microbiota plays such a significant role, many studies have focused on their role in different diseases and potential in disease diagnosis and prognosis assessment [19]. Animal studies showed that gut microbiota can be used to facilitate the diagnosis of liver cirrhosis in rats with good accuracy [20]. A clinical study showed that the content of *Faecali* bacterium decreased in patients with bipolar disorder and was closely related to the severity of the disease [21]. In addition, the ratio of different species can also be used in the diagnosis of some diseases; for example, the ratio of *Firmicutes/Bacteroidetes* is often cited as a marker of obesity [22]. Recent studies have showed that acute *S. japonicum* infection leads to alteration in gut microbiome composition [23]. In contrast, the gut microbiota in patients with *S. japonicum* infection-induced liver cirrhosis is similar to that in healthy control groups. However, gut microbiota signatures in patients with chronic and advanced *S. japonicum* infection remain unclear, and the potential role of gut microbiota in the progression of *S. japonicum* infection is still unknown. Thus, profiling gut microbiota in different stages of *S. japonicum* infection will not only deepen our understanding of the mechanism of *S. japonicum* infection in the aspects of gut microbiome, but also shed light on the potential role of gut microbiota as a non-invasive biomarker for diagnosis as well as prognostic analysis of different stages of *S. japonicum* infection.

In this study, we use 16S ribosomal RNA gene sequencing to profile the gut microbiota in patients with chronic and advanced *S. japonicum* infection. Revealing the features of intestinal microbial community with *S. japonicum* in different stages will contribute to further understanding of the pathogenesis of *S. japonicum* infection developing from chronic to advanced stage and identifying potential non-invasive biomarkers for the diagnosis and prognosis of *S. japonicum* infection.

## Methods

### Study design

The study was conducted at the Third People's Hospital of Hanshou County in Dongting Lake area, Hunan Province, China, from February 2021 to February 2022. In this study, all patients (≥ 18 years old) with *Schistosoma japonica* infection visiting the Third People's Hospital of Hanshou County from February 2021 to February 2022 who agreed to participate in this study were included. According to the inclusion and exclusion criteria, 24 patients with chronic *S. japonicum* infection and five patients with advanced *S. japonicum* infection were included in this study. The fecal specimens and clinical information of the patients with *S. japonicum* infection in the hospital were collected.

### Inclusion criteria for study subjects

According to the National Standardized Diagnostic Criteria for *S. japonicum* infection (WS261-2006) of the Ministry of Health of China, patients with advanced schistosomiasis should meet the following conditions.

(i) History of repeated or prolonged exposure to water in infected areas.
(ii) History of treatment for *S. japonicum* infection.
(iii) Positive ovum test in the patient's stool or positive serum immunological test.
(iv) Patients with liver fibrosis.
(v) The patient has one or more complications of splenomegaly, hypersplenism, portal hypertension or ascites.

### Exclusion criteria for study subjects

(i) The patient was not currently in an acute infection state.
(ii) The patient did not have a tumor.
(iii) The patient was not infected with hepatitis A,B,C,D and E viruses.
(iv) The patient was not infected with other parasites.

The basic information of the patients is shown in Table 1.

**Table 1:** Participant profile

Patient number	Gender (male/female)	Age (years)	Diagnosis (advanced/chronic)
01	Male	66	Advanced
02	Male	53	Advanced
03	Male	67	Advanced
04	Female	71	Advanced
05	Female	58	Advanced
06	Male	69	Chronic
07	Male	63	Chronic
08	Male	52	Chronic
09	Female	55	Chronic
10	Female	70	Chronic
11	Female	55	Chronic
12	Female	58	Chronic
13	Male	55	Chronic
14	Male	50	Chronic
15	Male	62	Chronic
16	Male	61	Chronic
17	Male	54	Chronic
18	Male	45	Chronic
19	Female	68	Chronic
20	Female	55	Chronic
21	Female	50	Chronic
22	Male	49	Chronic
23	Male	59	Chronic
24	Male	46	Chronic
25	Male	50	Chronic
26	Male	50	Chronic
27	Male	51	Chronic
28	Male	46	Chronic
29	Male	65	Chronic

There were no statistically significant differences in gender and age between the two groups of patients. Differences in age were determined using the t-test, *t*_(27)_ = 0.389, *P* = 0.0698. Differences in gender were determined using the Chi-square test, *χ*2 = 0.2269*, df* = 1, *P* = 0.6338

### Sample collection

Study participants were trained by healthcare workers. Fresh stool samples were packed in sterile stool collection tubes. All specimens were snap-frozen in liquid nitrogen within half an hour of collection, transferred to sterile Eppendorf tubes and stored in a −80 °C freezer until DNA extraction was performed [24].

### Sequencing

#### Extraction of genome DNA

Total genome DNA from samples was extracted using CTAB method [25]. Magnetic Soil and Stool DNA Kit (TIANGEN DP712) was used. DNA concentration and purity were monitored on 1.0% agarose gels. According to the concentration, DNA was diluted to 1 ng/μl using sterile water.

#### Amplicon generation

Primer 16S V3-V4: 341F (5′-CCTACGGGNGGCWGCAG-3′) and 806R (5′-GGACTACHVGGGTATCTAAT-3′) 16S rRNA genes were amplified using the specific primer with the barcode. All PCR reactions were carried out in 30-μl reactions with 15 μl Phusion^®^ High-Fidelity PCR Master Mix (New England Biolabs), 0.2 μM forward and reverse primers and about 10 ng template DNA. Thermal cycling consisted of initial denaturation at 98 ℃ for 1 min, followed by 30 cycles of denaturation at 98 ℃ for 10 s, annealing at 50 ℃ for 30 s and elongation at 72 ℃ for 60 s and finally 72 ℃ for 5 min [25].

#### PCR product quantification and qualification

The same volume of 1× loading buffer (contained SYB green) was mixed with PCR products and electrophoresis operated on 2.0% agarose gel for detection. Samples with a bright main strip between 400 and 450 bp were chosen for further experiments.

#### PCR product mixing and purification

PCR products were mixed in equidense ratios. Then, the mixture of PCR products was purified with AxyPrep DNA Gel Extraction Kit (AXYGEN).

#### Library preparation and sequencing

Sequencing libraries were generated using NEB Next^®^ Ultra™ DNA Library Prep Kit for Illumina (NEB, USA) following the manufacturer’s recommendations, and index codes were added. Finally, the library was sequenced on an Illumina Miseq/HiSeq 2500 platform, and 250 bp/300 bp paired-end reads were generated [26].

### Data analysis

#### Paired-end read assemblies

Paired-end reads from the original DNA fragments were merged using FLASH (http://ccb.jhu.edu/software/FLASH/).

#### OTU cluster and species annotation

Sequences analyses were performed with UPARSE software package (http://drive5.com/uparse/) using the UPARSE-OTU and UPARSE-OUT ref algorithms. In-house Perl scripts were used to analyze alpha (within samples) and beta (among samples) diversity. Sequences with ≥ 97.0% similarity were assigned to the same OTUs [27]. We picked a representative sequence for each OTU and used the RDP classifier to annotate taxonomic information for each representative sequence.

#### Analysis

Chao1, ACE, Simpson and Shannon diversity indices were calculated by QIIME [28], and comparisons between groups were performed by t-test. Beta diversity analysis was visualized using PCA, PCoA and NMDS [20]. To confirm differences in the abundances of individual taxonomy between the two groups, STAMP software was utilized [29]. LEfSe (http://huttenhower.sph.harvard.edu/lefse/) was used for the quantitative analysis of biomarkers within different groups [30]. To identify differences of microbial communities between the two groups, ANOSIM was performed based on the Bray-Curtis dissimilarity distance matrices [31].

## Results

### Species composition analysis.

**Fig. 1:**
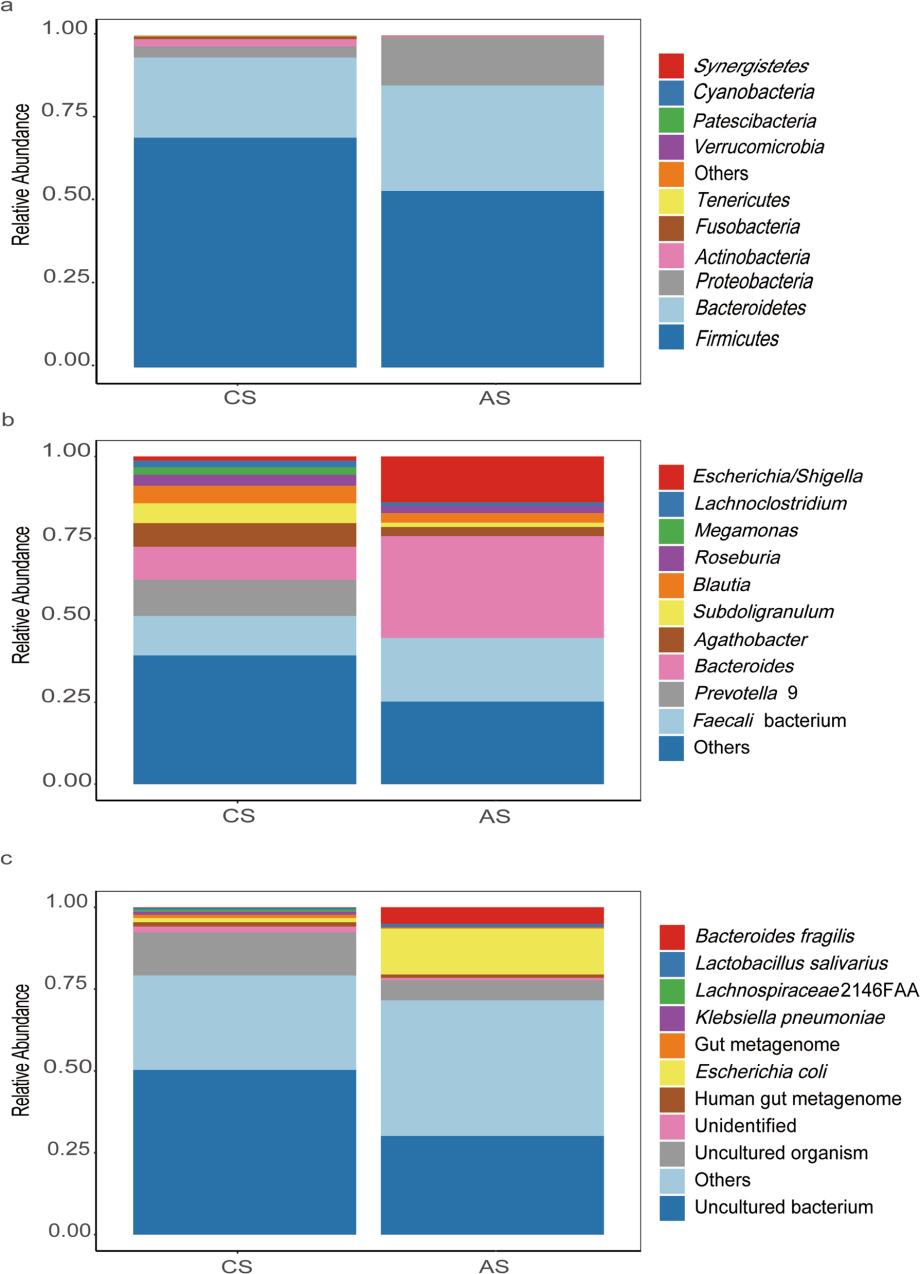
Species distribution of gut microbiota in patients with chronic and advanced schistosomiasis. Phylum level (**a**). Genus level (**b**). Species level (**c**). *CS* chronic *S. japonicum* infection, *AS* advanced *S. japonicum* infection

To explore the differences of gut microbiota between patients with chronic and advanced *S. japonicum* infection, we first analyzed the microbial composition of both groups of patients from three levels: phylum (Fig. 1a), genus (Fig. 1b) and species (Fig. 1c). We identified the top 10 species with differences at each level and their percentages. We found that at the phylum level, *Bacteroidetes* and *Firmicutes* were most abundant in both groups of patients. However, compared with patients with chronic *S. japonicum* infection, patients with advanced infection had a lower percentage of *Firmicutes* and a higher percentage of *Proteobacteria* (Fig. 1a). At the genus level, the percentages of *Faecalis* bacterium and *Bacteroides* were increased and the content of *Prevolla* 9 was significantly decreased in patients with advanced *S. japonicum* infection (Fig. 1b). *Escherichia coli* and *Bacteroides fragilis* were significantly increased in patients with advanced *S. japonicum* infection at species level (Fig. 1c). Thus, the gut microbiota structure altered when chronic *S. japonicum* infection progressed to advanced *S. japonicum* infection.

### Analysis of alpha and beta diversity of gut microbiota

**Fig.2:**
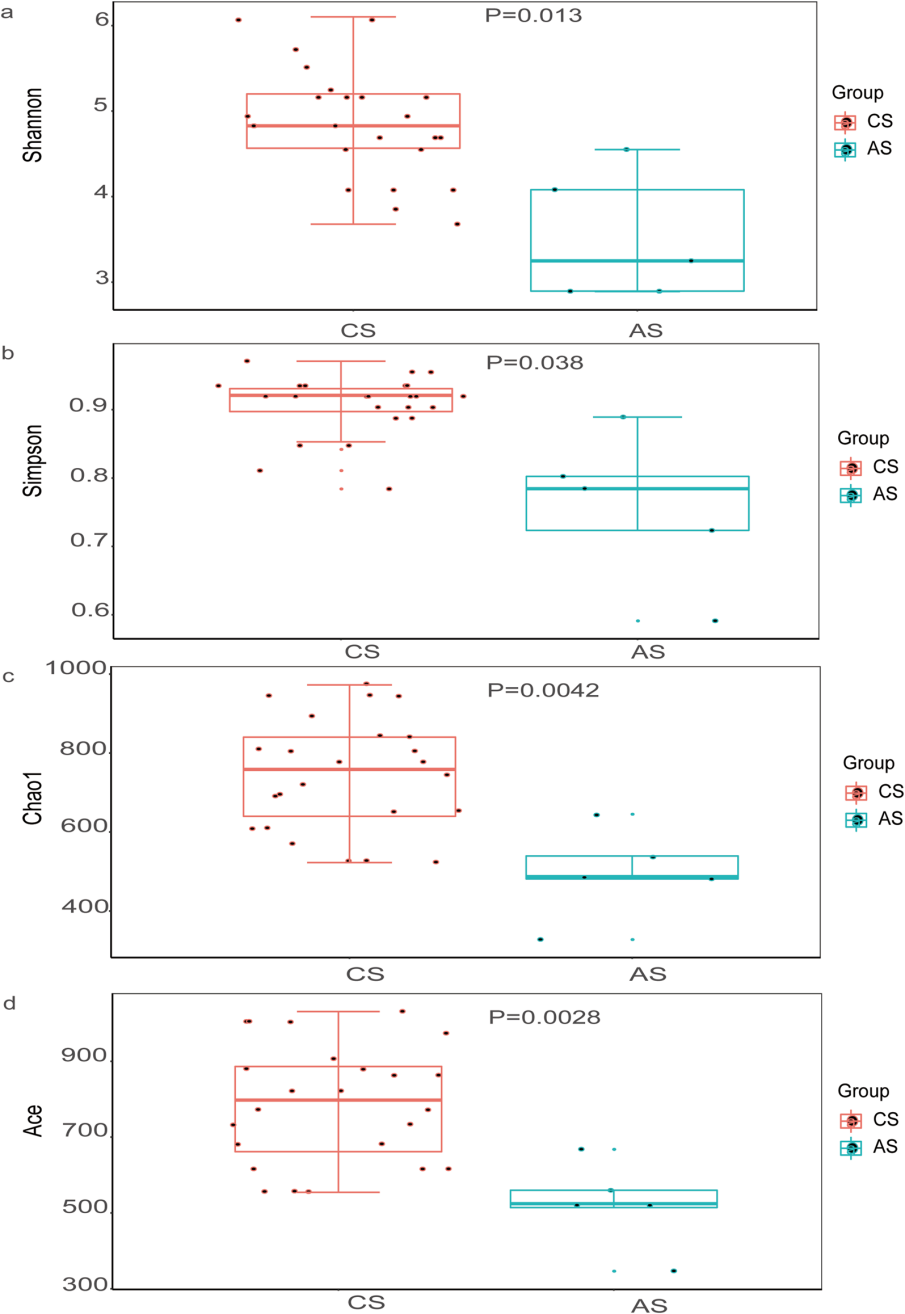
Alpha diversity analysis of intestinal flora in patients with chronic and advanced *S. japonicum* infection. The sequencing data were evaluated by four metrics: Shannon index (t-test, *t*_(27)_ = 2.05, *P* = 0.013) (**a**); Simpson index (*t*-test, *t*_*(27)*_ = 2.05, *P* = 0.038) (**b**); Chao 1 index (*t*-test, *t*_*(27)*_ = 2.77, *P* = 0.0042) (**c**); ACE index (*t*-test, *t*_(27)_ = 2.77, *P* = 0.0028) (**d**). *CS* chronic *S. japonicum* infection, *AS* advanced *S. japonicum* infection

**Fig.3:**
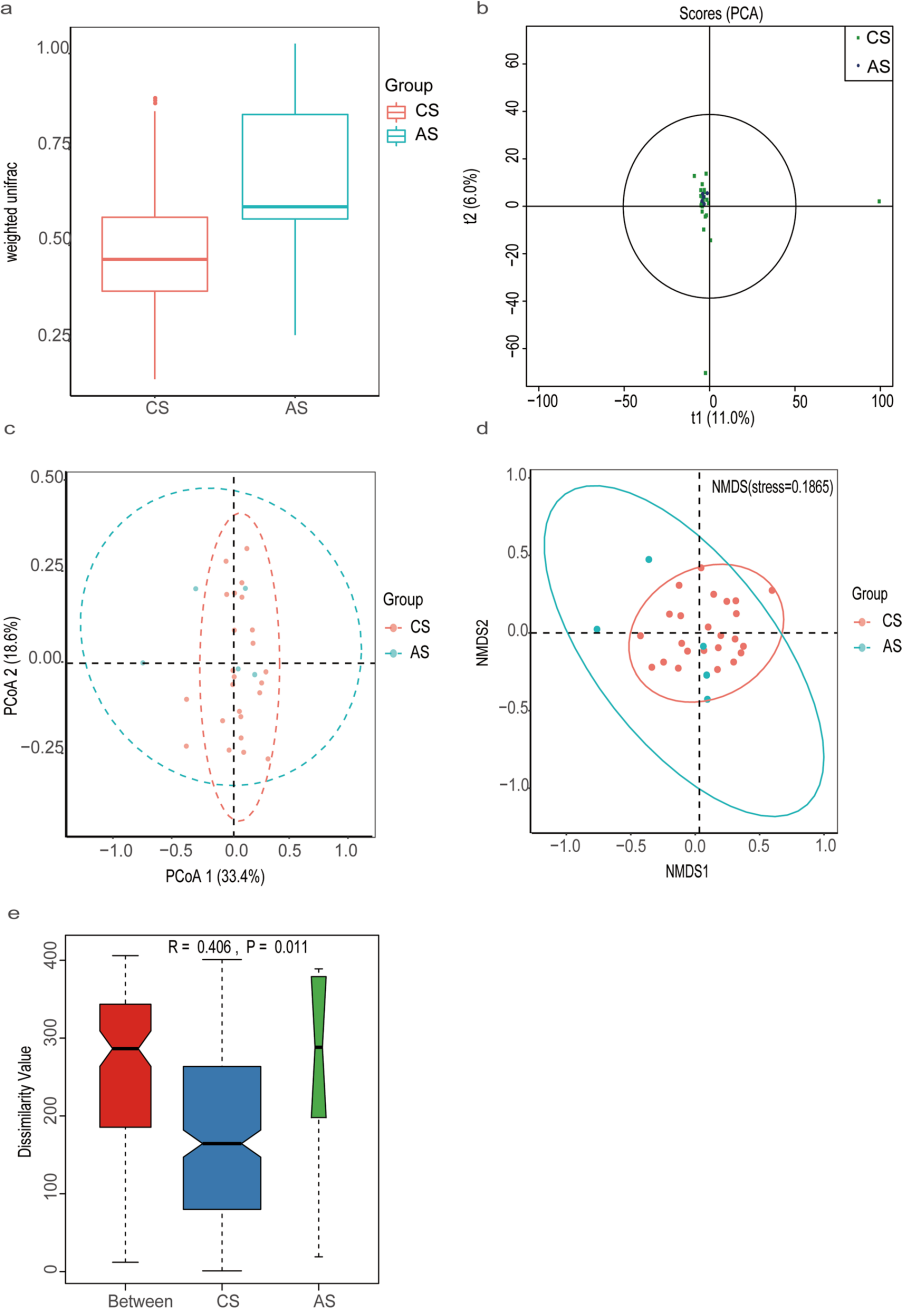
Beta diversity of intestinal flora in patients with chronic and advanced *S. japonicum* infection was analyzed based on weighted UniFrac (Wilcoxon, *Z* = *−*2.44, *P* = 0.0063) (**a**). PCA (**b**), PCoA (**c**) and NMDS analysis (**d**) are shown. Anosim analysis (*R* = 0.406; *P* = 0.011) (**e**). *CS*, chronic *S. japonicum* infection, *AS* advanced *S. japonicum* infection, *PCA* principal component analysis, *PcoA* principal coordinate analysis, *NMDS* non-metric multidimensional scaling, *ANOSIM* analysis of similarities

The results of Shannon index analysis showed that all samples were saturated (Additional file 1: Fig.S1), indicating that the amount of sequencing data was reasonable, the number of OTU species in the samples was sufficient, and the average abundance of different species reached the standard. For comparison of the bacterial community within groups, we used four metrics to analyze the data, including the Shannon(*t*-test,*t*_(27)_ = 2.05, *P* = 0.013)(Fig. 2a), Simpson(*t*-test, *t*_(27)_ = 2.05, *P* = 0.038) (Fig. 2b), Chao 1 (*t*-test, *t*_(27)_ = 2.77, *P* = 0.0042) (Fig. 2c) and ACE (*t*-test, *t*_(27)_ = 2.77, *P* = 0.0028) (Fig. 2d). All metrics suggested that there were significant differences in the bacterial community within groups. Patients with advanced *S. japonicum* infection had lower levels of microbial diversity and microbial abundance compared with patients with chronic *S. japonicum* infection. We further compared the bacterial community between the two groups using beta diversity analysis. Difference in beta diversity between patients with advanced and chronic *S. japonicum* infection was significant based on weighted Unifrac (Wilcoxon, *Z* = *−*2.44, *P* = 0.0063) (Fig. 3a). In addition, we performed PCA (Fig. 3b), PCoA (Fig. 3c) and NMDS analysis (Fig. 3d). To determine whether the grouping of this study was meaningful, we used ANOSIM analysis (based on the Bray-Curtis algorithm) to test whether the difference between groups was significantly greater than the difference within groups. ANOSIM analysis (based on the Bray-Curtis algorithm) demonstrated that the difference between the two groups of patients was greater than the difference within groups (*R* = 0.406; *P* = 0.011), and the statistical results were credible (Fig. 3e). Therefore, there are differences in the composition of gut microbiota between patients with advanced and chronic *S. japonicum* infection, and the diversity and abundance of intestinal flora in patients with advanced *S. japonicum* infection are lower than those in patients with chronic *S. japonicum* infection.

### Discovery of potential diagnostic biomarkers

**Fig. 4:**
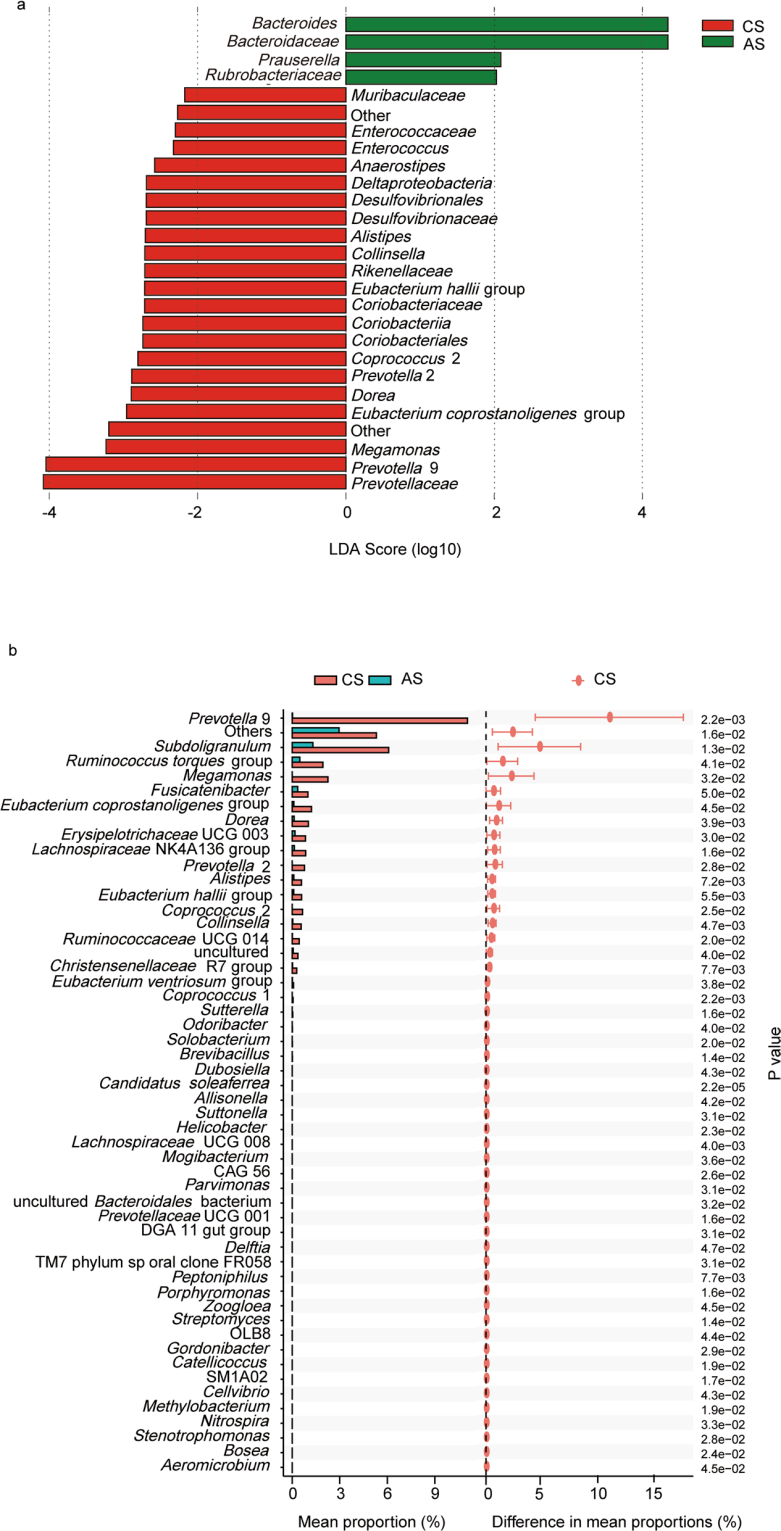
Species-specific differences in the gut microbiota between the groups. The histogram of LDA scores compared the two groups of patients with significant differences in the abundance of intestinal flora (**a**). The result of stamp analysis was shown (**b**). *CS* chronic *S. japonicum* infection, *AS* advanced *S. japonicum* infection, *LDA* linear discriminant analysis

To explore the species-specific differences in the gut microbiota between the groups of advanced and chronic *S. japonicum* infection and to find potential diagnostic markers, we next performed LEfSe analysis on the gut microbiota of the two groups of patients. The LEfSe analysis showed that there were differences in the intestinal flora between the two groups of patients at different species classification levels. At the family level, the dominant flora in patients with advanced *S. japonicum* infection was *Bacteroidaceae*; however, the *Prevotellaceaein* only existed in patients with chronic *S. japonicum* infection. At the genus level, the specific flora of the two groups of patients were *Bacteroides* and *Prevotella* 9 (Fig. 4a). In addition, we performed a stamp analysis on the intestinal flora of the two groups of patients at the genus level. The stamp analysis showed that the top five species with the highest percentage of differences between the two groups of patients were *Prevotella* 9, *Subdoligranulum*, *Ruminococcus torques*, *Megamonas* and *Fusicatenibacter* (*P* < 0.05) (Fig. 4b). According to our results, some specific species in the gut of patients with advanced and chronic *S. japonicum* infection are significantly different, and these species have potential to act as non-invasive biomarkers for differentiation of different stages of *S. japonicum* infection.

### Function prediction

**Fig. 5:**
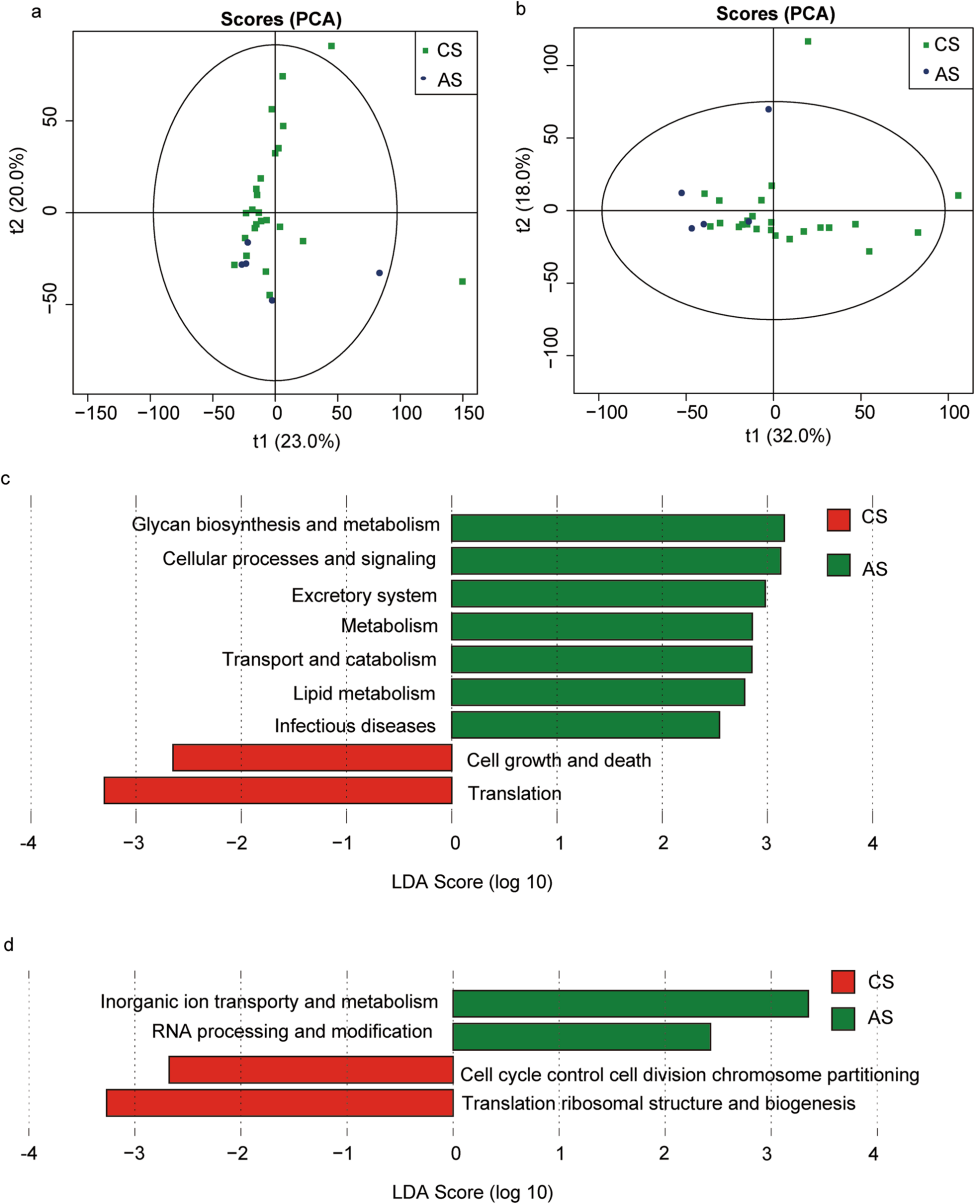
Prediction of intestinal flora function in patients with chronic and advanced *S. japonicum* infection. PCA analysis of two groups of patients based on the KEGG database (**a**). PCA analysis of two groups of patients based on the COG database (**b**). Histogram showing the difference in the abundance of gut bacterial function between the two groups of patients based on KEGG database (**c**). Histogram showing the difference in the abundance of gut bacterial function between the two groups of patients based on COG database (**d**). *CS* chronic *S. japonicum* infection, *AS* advanced *S. japonicum* infection, *COG* cluster of orthologous group, *KEGG* Kyoto Encyclopedia of Genes and Genomes

**Fig. 6:**
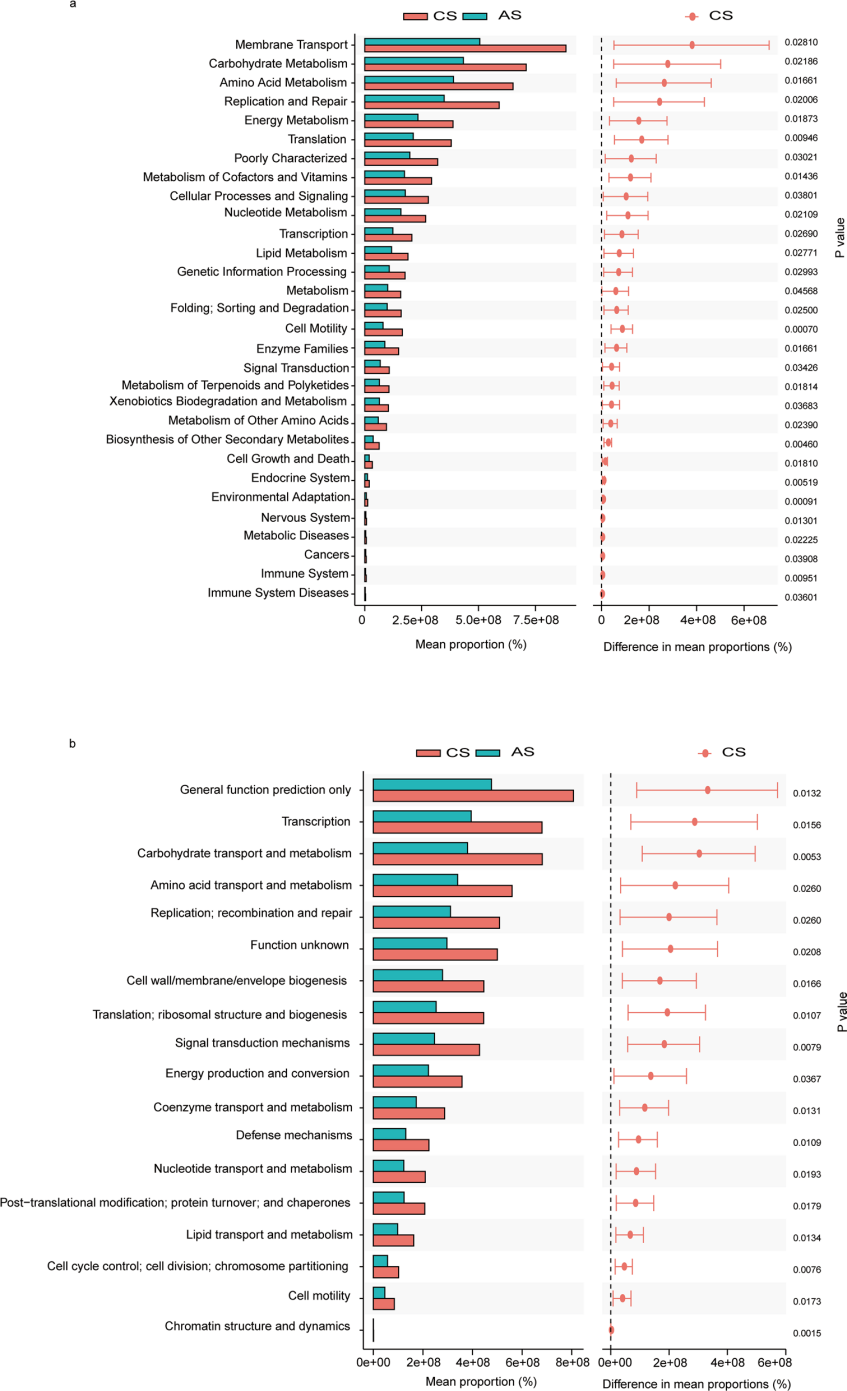
Stamp analysis on the functional differences between the two groups of patients. Result based on the KEGG database (**a**). Result based on the COG database (**b**). *CS* chronic *S. japonicum* infection, *AS* advanced *S. japonicum* infection, *COG* cluster of orthologous group, *KEGG* Kyoto Encyclopedia of Genes and Genomes

To explore the functional differences, we used the KEGG database and COG database to analyze the gut microbiota in two groups of patients. KEGG functional prediction analysis is an effective way to study the metabolic function changes of community samples to adapt to environmental changes. First, we applied PCA analysis to reveal the similarities or differences in the microbiota function of different groups at the overall level. The results of KEGG (Fig. 5a) and COG (Fig. 5b) suggested that there were differences in the function of the microbiota between the groups of patients with advanced and chronic *S. japonicum* infection. We used LEfSe analysis to further explore specific functional differences between the two groups. According to the KEGG database, the function of patients with chronic *S. japonicum* infection is mainly concentrated in translation and cell growth and death. In the group of patients with advanced *S. japonicum* infection, functional alterations mainly occurred in the elevation of metabolism-related functions (Fig. 5c). COG database analysis suggested the functional changes in patients with chronic *S. japonicum* infection were manifested in translation ribosomal structure and biogenesis and cell cycle control, cell division and chromosome partitioning. The functional changes in gut microbiota in the group of patients with advanced *S. japonicum* infection were mainly manifested as inorganic ion transport, metabolism, RNA processing and modification (Fig. 5d). We further performed a stamp analysis (Fig. 6a and b) on the functional differences between the two groups of patients, and the top five results are shown in Table 2.

**Table 2:** Significantly different flora functions

NO	Function	*P* value	Database (KEGG/COG)
1	Membrane transport	0.02810	KEGG
2	Carbohydrate metabolism	0.02186	KEGG
3	Amino acid metabolism	0.01661	KEGG
4	Replication and repair	0.02006	KEGG
5	Energy metabolism	0.01873	KEGG
6	Transcription	0.0156	COG
7	Carbohydrate transport and metabolism	0.0053	COG
8	Amino acid transport and metabolism	0.0260	COG
9	Replication; recombination and repair	0.0260	COG
10	Cell wall/membrane/envelope biogenesis	0.0166	COG

Welch’s *t*-test, *t*_(27)_ = 1.703, *P* < 0.05

## Discussion

*Schistosoma. japonicum* infection remains an important public health problem in the world, imposing a heavy social and economic burden on 78 countries in endemic areas. Big challenges must be overcomed to conquer this kind of parasitic disease as there is no effective vaccine and only praziquantel is used in the clinic. The mechanism of transition from chronic to advanced *S. japonicum* infection remains largely unknown and further study is needed. Evidences suggested that gut microbiota plays a role in the pathogenesis of *S. japonicum* infection and shows potential to act as a non-invasive biomarker in diagnosis and prognostic analysis. However, the composition of the gut microbiota in chronic and advanced *S. japonicum* infection is not clear. In this study, we for the first time compared the composition of the intestinal flora in patients with chronic and advanced *S. japonicum* infection. We found that alteration occurs in the gut microbiota between the groups of patients with chronic and advanced *S. japonicum* infection. Analysis of alpha diversity and beta diversity indicated that the diversity and abundance of intestinal flora in patients with advanced *S. japonicum* infection were lower than that in patients with chronic *S. japonicum* infection. *Prevotella* 9, *Subdoligranulum*, *Ruminococcus torques*, *Megamonas* and *Fusicatenibacter* could be biomarkers for diagnosis and help to discriminate different stages of *S. japonicum* infection. Besides, function prediction analysis revealed that microbiota function of the chronic group was focused on translation and cell growth and death, while that in the advanced group was concentrated on the elevation of metabolism-related functions. Our study demonstrated that alteration in gut microbiota in different stages of *S. japonicum* infection indicated a potential role of gut microbiota in the pathogenesis of transition from chronic to advanced *S. japonicum* infection. Furthermore, differently expressed bacteria seemed to have potential to facilitate differentiate diagnosis and prognosis analysis. However, further validation in the clinic is needed, and the underlying mechanism requires further study.

Gut microbiota alteration in different stages of *S. japonicum* infection was observed in our study. This was consistent with previous studies [24]. Numerous studies on liver disease have reported that it is closely related to gut microbes [32]. With the aggravation of liver disease, the alpha diversity of patients' gut microbiota decreased significantly [33]. This phenomenon was also observed in our study; patients with advanced *S. japonicum* infection had lower levels of gut microbiota alpha diversity than patients with chronic *S. japonicum* infection. Apart from alpha diversity, we also observed differences between individuals in each group. Although differences within groups will affect statistical analysis, ANOSIM analysis suggested that the difference between the two groups of patients was greater than the difference within the groups and the statistical results were reasonable and credible. In addition, we found that patients with advanced *S. japonicum* infection had higher beta diversity levels than patients with chronic *S. japonicum* infection. In another comparison between patients with *S. japonicum* infection-associated cirrhosis and healthy people, the researchers also found that patients with *S. japonicum* infection-associated cirrhosis had increased levels of beta diversity [34]. Although the different controls were chosen in our study, both studies demonstrate that *S. japonicum* infection and disease progression lead to increased beta diversity in the gut microbiota.

Due to the lack of effective diagnostic methods to differentiate patients with chronic *S. japonicum* infection from those with advanced *S. japonicum* infection in clinical practice, an important goal of our study was to explore whether there are potential biological targets for disease staging based on gut microbes. Our results showed that the content of *Bacteroides* in the intestine of patients with advanced *S. japonicum* infection was much higher than that of patients with chronic *S. japonicum* infection. *Bacteroides* is a double-edged sword to the human body [35]. Recent studies showed that elevated levels of *Bacteroides* can lead to the occurrence of colorectal tumors [36], consistent with the increased incidence of colorectal cancer in patients with advanced *S. japonicum* infection [37]. Thus, *Bacteroides* not only has potential to act as a staging marker for *S. japonicum* infection, but also could be a potential marker for cancer risk assessment. However more studies are needed to validate this function. The most abundant microbiota in people with chronic *S. japonicum* infection is *Prevotella* 9, which helps break down proteins and carbohydrates. It can also act as an opportunistic pathogen to induce intestinal [38] and vaginal inflammation [39]. Patients with chronic *S. japonicum* infection have been in a chronic inflammatory state for a long time. The increase in the content of *Prevotella* in the intestine is related to the reported changes in intestinal flora in other inflammatory states. Although numerous studies have provided sufficient evidences for its association with inflammation, currently there are insufficient data to confirm the causal relationship between *Prevotella* and the inflammatory state in patients with chronic *S. japonicum* infection.

After discovering the differences in intestinal flora between patients with chronic and advanced *S. japonicum* infection, we further explored the possible differences in physiological functions between the two groups of patients due to differences in gut microbes. The results of the KEGG and COG databases suggested that metabolism-related gene expression changed significantly in patients with advanced *S. japonicum* infection, while in patients with chronic *S. japonicum* infection, it was mainly the expression of cell cycle and cell death-related genes, consistent with the development of *S. japonicum* infection. The liver and intestinal tissues of patients with chronic *S. japonicum* infection are damaged [3]. However, organ function can still be compensated, so the function of patients during this period is mainly focused on tissue damage, repair and cell death. As the disease progresses, the patient will transit into advanced stage. When many tissues and cells within the liver and intestinal tract develop necrosis or apoptosis, tissue fibrosis occurs [40]. The liver and intestine are the most important digestive and metabolic organs, also as the target organs of *S. japonicum* infection. When the damage is too serious, it leads to disorders of the metabolism and immune system [41]. This also explains the increased incidence of metabolic and infectious diseases in patients with advanced *S. japonicum* infection. When the disease continues to progress, due to insufficient energy supply and application, metabolic disorders occur, and then patients become weaker, gradually lose their ability to work [42] and even die.

In recent years, because of the efficient and standardized treatment for patients with *S. japonicum* infection in China, new cases of *S. japonicum* infection have been dramatically reduced. The infection in many chronic patients has been stopped from progressing to advanced stage; meanwhile, the number of patients with advanced *S. japonicum* infection has significantly decreased [43]. Many of the patients with pneumonia, cholecystitis or hepatitis B were excluded, as these diseases affect the composition of the gut microbiota [44–46]. Therefore, few patients with advanced *S. japonicum* infection were included in this study. Although the limited patient number in the advanced group was a weakness of our study, ANOSIM analysis suggested that the within-group difference was acceptable and the statistical results reliable. Of course, an increased number of patients in advanced group would definitely improve the study, and more validation in the future is needed. Although we found some microbiota with potential as biomarkers to distinguish patients with chronic *S. japonicum* infection from those with advanced *S. japonicum* infection, more research is needed to clarify the causal relationship between different species and disease progression. The functional changes caused by alterations in the gut microbiota also need further verification in the clinic.

## Conclusions

By using 16S rRNA sequencing technology, our study found that there were differences in gut microbiota between patients with chronic and advanced *S. japonicum* infection. Patients with advanced *S. japonicum* infection had lower alpha and higher beta diversity when compared with patients with chronic *S. japonicum* infection. The proportion of *Bacteroides* in the intestine of patients with advanced schistosomiasis was higher, while the content of *Prevotella*, especially *Prevotella* 9, was relatively abundant in patients with chronic *S. japonicum* infection. Thus, *Prevotella* 9 has the potential to become a biomarker to distinguish patients with chronic *S. japonicum* infection from patients with advanced *S. japonicum* infection. The functions of flora in patients with chronic *S. japonicum* infection are mainly focused on the cell cycle and cell death, while those in patients with advanced *S. japonicum* infection are mainly focused on metabolism.

## Supplementary Information

**Additional file 1: Fig. S1.** Shannon curve showed that all samples were saturated.

## Data Availability

Data supporting the conclusions of this article are included within the article.

## Abbreviations

ANOSIM: Analysis of similarities
16S rRNA: 16S ribosomal ribonucleic acid
COG: Cluster of orthologous group
CTAB: Cetyltrimethylammonium bromide
DNA: Deoxyribonucleicacid
KEGG: Kyoto Encyclopedia of Genes and Genomes
LDA: Linear discriminant analysis
LEfSe: Linear discriminant analysis effect size
NMDS: Non-metric multidimensional scaling
OUT: Operational taxonomic unit
PCR: Polymerase chain reaction
PCA: Principal component analysis
PCoA: Principal coordinate analysis
*S. japonicum *: *Schistosoma japonicum*
