# Supplementary figures and images for: Comparison of intestinal flora between patients with chronic and advanced Schistosoma japonicum infection
Source: Parasit Vectors. 2022 Nov 7;15:413. doi: 10.1186/s13071-022-05539-6 (PMC9640844; doi:10.1186/s13071-022-05539-6)

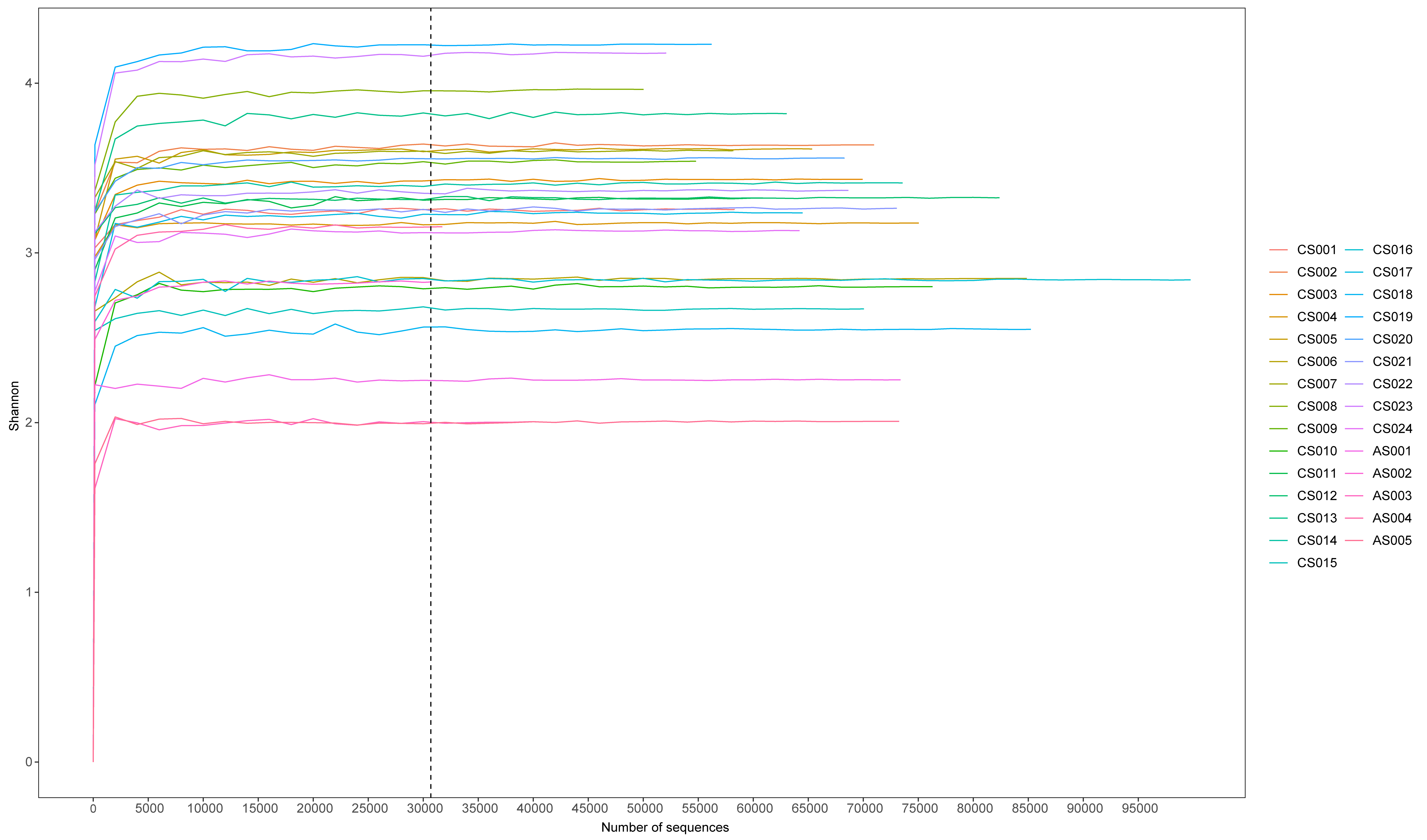

Supplement: Supplementary file 1 — Additional file 1: Fig. S1. Shannon curve showed that all samples were saturated. [file 13071_2022_5539_MOESM1_ESM.tif]
